# Panoramic Soft Tissue Calcifications as Vascular Risk Markers in Diabetes: A Pilot Case–Control Study in Hungarians

**DOI:** 10.1016/j.identj.2026.109584

**Published:** 2026-05-09

**Authors:** Laura Lipták, Klaudia Lipták, Péter Hermann, Noémi Katinka Rózsa, Ádám Végh, Dorottya Bányai, Béla Kolarovszki, Gyula Marada, Dániel Végh, Attila Mühl

**Affiliations:** a Department of Paediatric Dentistry and Orthodontics, Diabetes Dental Working Group, Semmelweis University, Budapest, Hungary; b Department of Prosthodontics, Diabetes Dental Working Group, Semmelweis University, Budapest, Hungary; c Medical Faculty, Dental School, University of Pécs, Pécs, Hungary; d Department of Oral Diagnostics, Diabetes Dental Working Group, Semmelweis University, Budapest, Hungary

**Keywords:** Carotid artery calcification, Panoramic radiography, Diabetes mellitus, Soft tissue calcification, Stylohyoid ligament, Vascular risk

## Abstract

**Introduction:**

Soft tissue calcifications on dental panoramic radiographs (PR) may reflect systemic vascular disease. Given the increased cardiovascular and cerebrovascular risk in diabetes, this study compared their prevalence in patients with diabetes and baseline-comparable nondiabetic controls and explored associations in the combined sample.

**Methods:**

This retrospective case–control study included 56 adults with diabetes and 56 nondiabetic controls, frequency-matched by age (±5 years) and sex. Four calibrated examiners independently evaluated PRs with consensus adjudication. Outcomes were soft tissue calcification, carotid-region calcification, and stylohyoid ligament complex calcification/ossification. Crude prevalence was calculated for each group and the total sample, and multivariable logistic regression adjusted for age, sex, diabetes status, smoking, alcohol use, renal disease, and rheumatic disease.

**Results:**

A total of 112 participants were analysed (56 with diabetes mellitus and 56 controls), with comparable baseline characteristics between groups. In the total sample, the crude prevalence of any soft tissue calcification was 42.0%, carotid-region calcification 27.7%, and stylohyoid ligament complex calcification/ossification 35.7%. In adjusted models across the combined sample, age was independently associated with higher odds of any soft tissue calcification (per 10-year increase: aOR 1.68, 95% CI 1.23-2.29; *P* = 0.001) and carotid-region calcification (aOR 1.52, 95% CI 1.08-2.14; *P* = .016). Male sex was associated with lower odds of any soft tissue calcification (aOR 0.40, 95% CI 0.16-0.99; *P* = .047). Diabetes status was not independently associated with the outcomes in the fully adjusted models.

**Conclusion:**

Panoramic soft tissue calcifications were common. Although diabetes was not an independent predictor, frequent detection of suspected carotid-region calcifications supports structured reporting and opportunistic vascular risk assessment on routine PR, especially in older patients.

**Clinical relevance:**

Opportunistic identification of carotid-region calcifications on routine PR may support early cardiovascular risk assessment, particularly in patients with diabetes and older adults.

## Introduction

Diabetes mellitus is a prevalent chronic disease associated with both microvascular and macrovascular complications, including ischaemic stroke and cardiovascular disease.^1^^,^^2^ People with diabetes have around a 1.5 to 2-fold higher risk of stroke than individuals without diabetes, and they experience poorer functional outcomes after cerebrovascular events.^1^ In parallel, a substantial body of evidence has established a bidirectional relationship between diabetes and oral health: chronic hyperglycaemia adversely affects periodontal and peri-implant tissues, while periodontitis and other oral inflammatory conditions may worsen glycaemic control.^3^, ^4^, ^5^ These links position the dental setting as a promising environment for opportunistic screening and risk stratification in people living with diabetes.

The Semmelweis University Diabetes Dental Working Group and others have highlighted how dental professionals can contribute to diabetes care by integrating chairside metabolic assessment and structured counselling into routine practice. Preoperative measurement of HbA1c and blood glucose has been advocated before oral surgery and implant therapy in patients with known or suspected diabetes as a simple means to reduce peri-operative risk and identify undiagnosed dysglycaemia.^6^ International and Hungarian surveys have also shown that people with type 1 and type 2 diabetes often have limited oral-health knowledge and suboptimal oral-hygiene behaviours, underscoring the need for closer collaboration between dentistry and diabetology.^7^ Within this framework, radiographic findings obtained during routine dental care may provide additional, yet largely underused, information about systemic vascular risk.

Panoramic radiography (PR) is routinely performed in general and specialist dental practice because it provides a broad overview of the maxilla, mandible, and adjacent cervical and paranasal structures in a single exposure. Large retrospective series have shown that PRs frequently reveal incidental soft-tissue calcifications in the head and neck region.^8^, ^9^, ^10^, ^11^, ^12^, ^13^ These radiopacities include antroliths, tonsilloliths, sialoliths, calcified lymph nodes, phleboliths, mucous retention pseudocysts, calcified or ossified triticeous and thyroid cartilages, calcifications along the stylohyoid ligament complex, and calcifications in the region of the carotid artery bifurcation. Contemporary atlases and educational schemata group these entities according to their typical anatomic location on PRs, which facilitates systematic reporting in clinical and research settings. Our study focuses on panoramic soft tissue calcifications, with carotid-region and stylohyoid ligament calcifications as predefined key subtypes.

Reported prevalences of soft-tissue calcifications on PRs vary widely between populations. In mixed dental cohorts, overall prevalence rates between approximately 5% and 40% have been described, depending on age distribution, inclusion criteria, and whether stylohyoid ligament ossification is counted as a soft-tissue calcification.^8^, ^9^, ^10^, ^11^, ^12^, ^13^ Studies focusing on older adults or edentulous patients typically show higher rates and a greater number of calcification types per patient.^10^^,^^11^ Stylohyoid ligament calcification or elongation, tonsilloliths, and calcifications in the carotid region are consistently among the most frequently observed entities, whereas calcified lymph nodes, sialoliths, and phleboliths are less common.^9^, ^10^, ^11^, ^12^, ^13^, ^14^ Although many of these calcifications are asymptomatic and discovered by chance, their recognition is important to avoid misdiagnosis and to identify individuals who may require further evaluation.

Ossification or calcification of the stylohyoid ligament complex and elongation of the styloid process may be associated with cervicofacial pain, dysphagia, and other symptoms collectively referred to as Eagle syndrome.^15^, ^16^, ^17^ PRs represent a useful first-line tool for detecting elongated or mineralised stylohyoid structures, even though cross-sectional imaging may be necessary to delineate their exact extent and relationship to neighbouring neurovascular structures.^15^, ^16^, ^17^ Calcified lymph nodes can reflect previous or ongoing chronic infection, whereas vascular calcifications in the cervical region usually indicate underlying atherosclerotic disease.^8^^,^^9^^,^^12^^,^^13^ The ability to differentiate among these entities on PR is therefore essential for both diagnostic accuracy and appropriate referral.

Particular attention has been paid to calcifications located inferolateral to the mandibular angle at the approximate level of the third and fourth cervical vertebrae, which may represent calcified carotid artery atheroma. A series of classic and more recent studies has demonstrated that such carotid artery calcifications (CACs) visible on PRs are associated with ultrasonographically confirmed carotid plaque and stenosis and may predict future cardiovascular and cerebrovascular events.^18^, ^19^, ^20^, ^21^ In patients with type 2 diabetes mellitus, Friedlander and colleagues reported markedly higher prevalences of CACs—around 20%—on PRs compared with approximately 4% in age- and sex-matched nondiabetic controls.^18^^,^^19^ Ezoddini-Ardakani et al. subsequently confirmed, in an Iranian cohort, that calcified carotid atheromas were significantly more frequent among patients with type 2 diabetes than among nondiabetic subjects.^20^ More recent population-based and clinic-based studies have linked CACs on PR not only to diabetes but also to systemic conditions such as hypertension, chronic kidney disease, and established cardiovascular disease, further supporting their value as markers of generalised atherosclerosis.^21^^,^^22^

Beyond isolated carotid findings, emerging evidence suggests that people with diabetes may exhibit a broader pattern of ectopic calcification detectable on dental radiographs. A recent retrospective observational study reported that both carotid artery calcifications and pulp stones were significantly more common in diabetic patients than in nondiabetic controls on PRs, with relative risks in the range of 1.8 to 2.6.^23^ In another investigation, the overall burden of soft-tissue calcifications on digital PRs was associated with systemic diseases, including diabetes and hypertension, suggesting that such calcifications may serve as easily observable markers of systemic risk.^14^ However, most available studies have either focused narrowly on CACs or evaluated heterogeneous dental populations without detailed metabolic characterisation. Data on the prevalence and pattern of soft-tissue calcifications in well-defined cohorts of patients with diabetes—particularly those managed in specialised diabetes–dental collaborations—remain limited.

The Semmelweis University Diabetes Dental Working Group provides comprehensive dental care for adults with type 1 and type 2 diabetes and has previously reported on perioperative metabolic assessment and on oral-health-related behaviours in this population.^6^^,^^7^^,^^24^ Within this clinical framework, PRs obtained as part of routine diagnostics offer a unique opportunity to explore whether radiographically detectable soft-tissue calcifications are more prevalent among patients with diabetes than among nondiabetic dental patients, and whether particular anatomical patterns—such as calcifications in the carotid region or along the stylohyoid ligament—are especially frequent in this high-risk group.

Therefore, the primary aim of the present retrospective case–control study was to compare the prevalence of calcifications in the carotid artery region, as visualised on PRs, between patients with and without diabetes mellitus treated in a university dental setting. Our primary hypotheses were: H₀, there is no difference in the prevalence of carotid-region calcifications between patients with and without diabetes; and H₁, calcifications in the carotid region are more prevalent among patients with diabetes. As a secondary aim, we sought to describe the distribution of different soft tissue calcification types detectable on panoramic radiographs, including carotid-region calcifications and calcification or ossification of the stylohyoid ligament complex, and to explore their associations with demographic and clinical variables. By doing so, we aim to highlight the opportunistic information that may be obtained from routinely acquired dental PRs, without implying their use as a primary diagnostic tool for cerebrovascular disease.

Panoramic radiographs (PRs) are not a gold-standard vascular imaging modality; rather, they are routinely acquired for dental indications. Several studies have shown that calcifications in the carotid bifurcation region visible on PRs correlate with ultrasonographically confirmed carotid plaque and stenosis and may reflect generalised atherosclerotic burden.^21^^,^^25^ In line with current radiological recommendations that all visualised structures should be systematically evaluated, our study focuses on the opportunistic diagnostic potential of PRs for incidental soft tissue calcifications, rather than on PR as a primary vascular imaging technique.

## Materials and methods

### Study design and ethical approval

This retrospective case–control study was conducted at the Department of Prosthodontics, Semmelweis University (Budapest, Hungary), within the framework of the Diabetes Dental Working Group. Data were obtained from 2 existing anonymised clinical databases: (1) the retrospective database of adult patients with diabetes mellitus treated in the Diabetes Dental Working Group, and (2) an anonymised database of nondiabetic patients treated at the same department.

The study protocol was approved by the institutional research ethics committee of Semmelweis University (approval number: RKEB: 204/2018) and conducted in accordance with the Declaration of Helsinki. Because only de-identified data and existing radiographs were used, the requirement for individual informed consent was waived.

### Study population and data sources

At the time of data extraction, 56 adult patients (≥18 years) with previously diagnosed diabetes mellitus (type 1 or type 2) had a digital PR and complete baseline medical information recorded in the Diabetes Dental Working Group database; all of these patients were eligible and were included as the diabetes group. Baseline variables available and used in the present analysis included age, sex, hypertension, cardiovascular disease, coagulation disorder, thyroid disease, rheumatic disease, pulmonary disease, renal disease, gastrointestinal disease, drug allergy, current smoking, and regular alcohol consumption. Diabetes-specific variables (type of diabetes, duration of diabetes, type of antidiabetic treatment, presence of diabetes-related complications, use of continuous glucose monitoring, and use of insulin pump therapy) were also recorded but are analysed only within the diabetes group.

The control group without diabetes mellitus was selected from an existing anonymised database of patients treated at the same department during the same time frame and who underwent digital PR for routine dental diagnostic purposes. Potential controls must have no documented diagnosis of diabetes mellitus and no self-reported diabetes at the time of dental treatment. For each patient with diabetes, at least 1 control without diabetes was selected using frequency matching by age (±5 years) and sex. Comorbidities were not used as formal matching variables; however, during control selection we intentionally aimed to include patients with broadly similar cardiometabolic profiles (e.g. hypertension, cardiovascular and renal disease), so that the primary difference between groups would be the presence or absence of diabetes mellitus.

Patients with PRs of insufficient image quality, incomplete visualisation of the mandibular angle and adjacent cervical region, or a history of major head and neck surgery that could substantially alter the regional anatomy were excluded from both groups. All data were exported from the clinical databases in anonymised form; names and direct identifiers were removed before analysis, and a unique study code was assigned to each participant.

### Radiographic acquisition

All PRs had been obtained as part of routine dental diagnostics using digital panoramic units available in the department. Examinations were performed by trained radiology technicians according to the manufacturer’s recommendations, with standardised patient positioning and exposure parameters. Only radiographs judged to be of sufficient diagnostic quality for evaluation of the mandibular angle region and adjacent cervical soft tissues were included.

### Radiographic evaluation and classification of calcifications

PRs were exported in DICOM or high-resolution JPEG format and viewed on calibrated monitors in a dimly lit room. Four examiners (dentists with experience in oral and maxillofacial radiology and prosthodontics) independently assessed all radiographs. Before the main evaluation, the examiners underwent a joint calibration session using representative example images to harmonise the visual criteria for the different calcification categories, based on an established anatomy-based radiographic scheme for head and neck soft tissue calcifications on PRs.^8^^,^^11^^,^^12^ The schematic panoramic illustration demonstrates the typical anatomical locations of various soft tissue calcifications using distinct color-coded markers. These include sialoliths (orange), phleboliths (pink), antroliths (yellow), tonsilloliths (green), lymph node calcifications (brown), thyroid/triticeous cartilage calcifications (purple), carotid artery calcifications (red), and stylohyoid ligament calcification (blue) (Figure 1).

**Fig. 1:**
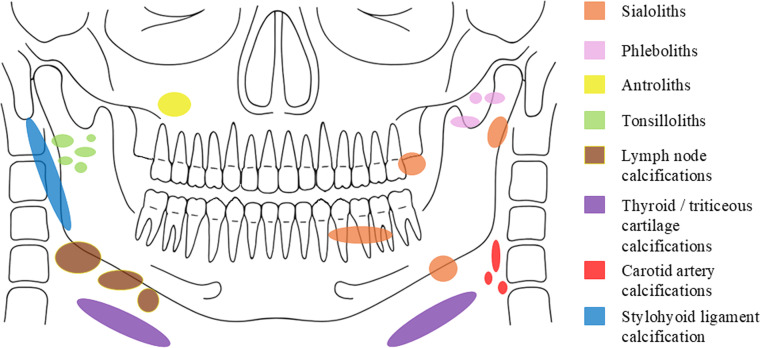
Schematic localisation of soft tissue calcifications on panoramic radiographs (PRs).

All images were anonymised and presented in random order; examiners were blinded to diabetes status and all clinical data. Using a standardised electronic data sheet, each examiner recorded:

1. Any soft tissue calcification within the panoramic field of view (primary radiographic variable; yes/no). This was scored as ‘yes’ if at least 1 soft tissue calcification compatible with the predefined categories was present anywhere in the head and neck region.
2. Carotid-region calcification (potential carotid plaque) (secondary variable; yes/no): radiopacities located inferolateral to the mandibular angle at the approximate level of the C3 to C4 vertebrae, projected over the cervical soft tissues, with nodular, verticolinear, or irregular morphology consistent with possible calcified carotid atheroma.
3. Stylohyoid ligament complex calcification (secondary variable; yes/no): linear, segmented, or nodular radiopacities extending from the temporal bone region toward the lesser horn of the hyoid bone, compatible with elongation or calcification/ossification of the stylohyoid ligament complex.

Other soft tissue calcification types (e.g., tonsilloliths, sialoliths, calcified lymph nodes, laryngeal cartilage calcifications, phleboliths) were also recorded and categorised according to the same anatomy-based scheme^8^^,^^11^^,^^12^ for descriptive purposes, but the primary analyses focus on the presence of any soft tissue calcification, with carotid-region and stylohyoid ligament calcifications treated as predefined key subtypes.

Based on the above, each of the 4 examiners evaluated all radiographs independently. Any discrepancies-even in outlier cases- were resolved by consensus. Where all 4 examiners agreed from the outset, the unanimous rating was accepted without modification.

Four examiners independently evaluated all panoramic radiographs on calibrated diagnostic monitors in a dimly lit room. Before the main readings, the examiners jointly reviewed a series of pilot cases to familiarise themselves with the classification scheme and to harmonise the distinction between carotid-region calcifications and other structures such as calcified laryngeal cartilages and the posterior horn of the hyoid bone. During this calibration phase, discrepant cases were discussed in detail and consensus decision rules were established. The subsequent readings were then performed in several batches; in each batch, all 4 examiners scored the presence or absence of (1) any soft tissue calcification, (2) carotid-region calcification and (3) stylohyoid ligament complex calcification/ossification on every radiograph. For each outcome, inter-examiner agreement was quantified on a randomly selected subset of panoramic radiographs using Fleiss’ kappa (κ). In the full dataset, any remaining discrepancies were resolved in a consensus session, and in these cases the final classification was determined by the board-certified dentomaxillofacial radiologist (AM).

### Outcome variables

The primary radiographic outcome of the study is the presence of any soft tissue calcification on the PR (yes/no).

The key secondary radiographic outcomes are:

1. Presence of carotid-region calcification (potential carotid plaque) (yes/no).
2. Presence of stylohyoid ligament complex calcification/ossification (yes/no).

For descriptive purposes, we also report the distribution of individual soft tissue calcification categories (e.g., stylohyoid ligament, tonsilloliths, vascular calcifications, calcified lymph nodes) as defined by the anatomy-based scheme.^8^^,^^11^^,^^12^

The main exposure variable is diabetes status (diabetes vs no diabetes). Diabetes-related characteristics (type of diabetes, duration, treatment, complications, continuous glucose monitoring, and insulin pump use) were analysed only within the diabetes group.

### Sample size calculation

The sample size calculation was based on the primary outcome, i.e., the presence of any soft tissue calcification on PRs, and on the expected difference in prevalence between patients with and without diabetes. Previous large retrospective studies in general dental populations have reported prevalences of soft tissue calcifications in the range of approximately 10% to 27%.^8^^,^^11^^,^^12^ Assuming that soft tissue calcifications would be more frequent in patients with diabetes than in nondiabetic controls, we conservatively hypothesised a prevalence of 33% in the diabetes group and 11% in the nondiabetic group.

Using these assumptions, a 2-sided chi-square test for comparison of 2 independent proportions, with an alpha level of 0.05 and a power of 80%, yielded a minimum required sample size of 54 participants per group. The available 56 patients with diabetes in our database, therefore, fulfil and slightly exceed this requirement, and at least an equal number of nondiabetic controls will be selected to satisfy the planned sample size.

### Statistical analysis

Baseline characteristics were summarised for the diabetes and nondiabetic control groups in the matched sample (n = 56 vs 56). Continuous variables are presented as mean ± standard deviation, whereas categorical variables are reported as counts and percentages. Between-group comparisons of baseline variables were performed using the independent-samples t-test for continuous data and Fisher’s exact test for categorical data, as appropriate.

Crude prevalence of the 3 radiographic outcomes was calculated for each group and the total study population (cases and controls combined; n = 112): any soft tissue calcification, carotid-region calcification (potential carotid plaque), and stylohyoid ligament complex calcification/ossification.

To assess independent associations between baseline factors and radiographic outcomes in the overall dataset, multivariable logistic regression models were fitted separately for each outcome in the combined sample (n = 112). The models included age, sex, diabetes status, current smoking, regular alcohol consumption, renal disease, and rheumatic disease. Age was modelled per 10-year increase. Results are reported as adjusted odds ratios (aORs) with 95% confidence intervals (CIs). All analyses were performed using R (R Foundation for Statistical Computing, Vienna, Austria) within the RStudio integrated development environment.^26^ A 2-sided *P*-value < .05 was considered statistically significant.

## Results

A total of 112 participants were included in the analysis, comprising 56 patients with diabetes and 56 nondiabetic controls.

Baseline sociodemographic and clinical characteristics are presented in Table 1. By design, the 2 groups were highly comparable: mean age was virtually identical (58.8 ± 16.5 vs 59.1 ± 16.6 years), and the proportion of male participants was the same in both groups (35.7% vs 35.7%). The prevalence of major cardiometabolic comorbidities was also very similar between patients with and without diabetes. Hypertension was common in both groups (62.5% vs 57.1%), as were cardiovascular disease (16.1% vs 16.1%) and renal disease (8.9% vs 8.9%), with no statistically significant between-group differences (Table 1).

**Table 1:** Baseline characteristics of patients with and without diabetes (matched sample, n = 56 vs 56).

Variable	Diabetes (n = 56)	Nondiabetic controls (n = 56)	*P-*value*
Sociodemographic variables			
Age, years	58.8 ± 16.5	59.1 ± 16.6	.94
Male sex	20 (35.7)	20 (35.7)	1.00
Comorbidities			
Hypertension	35 (62.5)	32 (57.1)	.70
Cardiovascular disease	9 (16.1)	9 (16.1)	1.00
Coagulation disorder	12 (21.4)	10 (17.9)	.81
Rheumatic disease	11 (19.6)	8 (14.3)	.62
Pulmonary disease	3 (5.4)	3 (5.4)	1.00
Renal disease	5 (8.9)	5 (8.9)	1.00
Gastrointestinal disease	10 (17.9)	11 (19.6)	1.00
Lifestyle factors			
Current smoking	5 (8.9)	4 (7.1)	1.00
Regular alcohol consumption	4 (7.1)	2 (3.6)	.68

⁎ *P*-values from independent-samples t-test (age) and Fisher’s exact test (categorical variables).

Other systemic conditions, including coagulation disorders, rheumatic disease, pulmonary disease, and gastrointestinal disease, showed comparable distributions across groups, and lifestyle factors were likewise balanced: current smoking (8.9% vs 7.1%) and regular alcohol consumption (7.1% vs 3.6%) were infrequent and did not differ significantly between patients with diabetes and controls. Overall, the close similarity of baseline characteristics suggests that confounding by the measured comorbidities is unlikely to account for subsequent differences in radiographic soft tissue calcification outcomes.

In the overall study sample of 112 participants (cases and controls combined), crude radiographic screening identified a substantial burden of soft tissue calcifications. Crude prevalence of each outcome was calculated in the diabetes and control groups and in the total sample. The prevalence of any soft tissue calcification was 42.0% (47/112). Potential carotid-region calcification was observed in 27.7% (31/112), while stylohyoid ligament calcification was present in 35.7% (40/112). These descriptive findings provide a cross-sectional snapshot of calcification frequency in the full dataset prior to group-wise or adjusted analyses (Table 2).

**Table 2:** Crude prevalence of panoramic radiographic calcification outcomes in the diabetes and control groups and in the total study population (n = 112).

Outcome	Diabetes (%)	Control (%)	Total (%)
Any soft tissue calcification	26/56 (46.4)	21/56 (37.5)	47/112 (42.0)
Carotid-region calcification (potential carotid plaque)	18/56 (32.1)	13/56 (23.2)	31/112 (27.7)
Stylohyoid ligament complex calcification/ossification	24/56 (42.9)	16/56 (28.6)	40/112 (35.7)

On the subset of panoramic radiographs used for reliability assessment, inter-examiner agreement for the presence of any soft tissue calcification was almost perfect (Fleiss’ κ = 0.91). Agreement for carotid-region calcification was substantial (κ = 0.73), and for stylohyoid ligament complex calcification/ossification it was almost perfect (κ = 0.86). These values indicate high inter-examiner reliability for all 3 radiographic outcomes.

Across the total study population (n = 112), multivariable logistic regression models were fitted for each outcome with adjustment for age, sex, diabetes status, smoking, regular alcohol consumption, renal disease and rheumatic disease. Figure 2 illustrates a representative PR from one of the study participants, shown both in its native form and with colour-enhanced annotations, on which the identified soft tissue calcifications are highlighted in distinct colours to enhance visualisation of their location and morphology. As shown in Figure 3, panel (a) shows bilateral calcifications located inferior to the hyoid bone and anterior to the cervical spine, consistent in location and morphology with suspected carotid-region atheromatous plaque. In panel (b), marked calcification and partial ossification of the stylohyoid ligament complex are visible, corresponding to the radiographic appearance typically associated with Eagle syndrome. Panel (c) demonstrates an example of another type of soft tissue calcification, with a phlebolith located anterior to the right temporomandibular joint condyle. Arrows indicate the location of the respective calcifications. For any soft tissue calcification, age was independently associated with higher odds (per 10-year increase: aOR 1.68, 95% CI 1.23-2.29; *P* = .001). Male sex showed lower odds compared with females (aOR 0.40, 95% CI 0.16-0.99; *P* = .047). Diabetes status was not independently associated with the presence of any soft tissue calcification in this fully adjusted model (aOR 1.55, 95% CI 0.67-3.55; *P* = .303). Smoking and regular alcohol use were not significant predictors. A statistically significant inverse association was observed for renal disease (aOR 0.17, 95% CI 0.03-0.94; *P* = .042), which should be interpreted cautiously given the small number of renal cases and the risk of sparse-data bias.

**Fig. 2:**
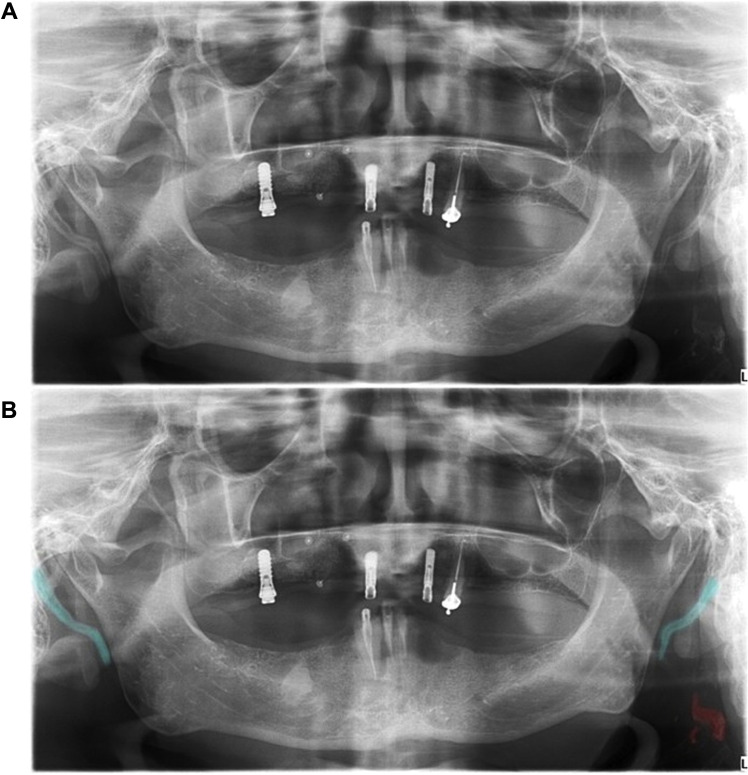
Native and colour-annotated panoramic radiograph (PR). A, Native image. B, Colour-enhanced image with blue indicating stylohyoid ligament calcifications and red indicating suspected carotid artery plaque-like calcifications.

**Fig. 3:**
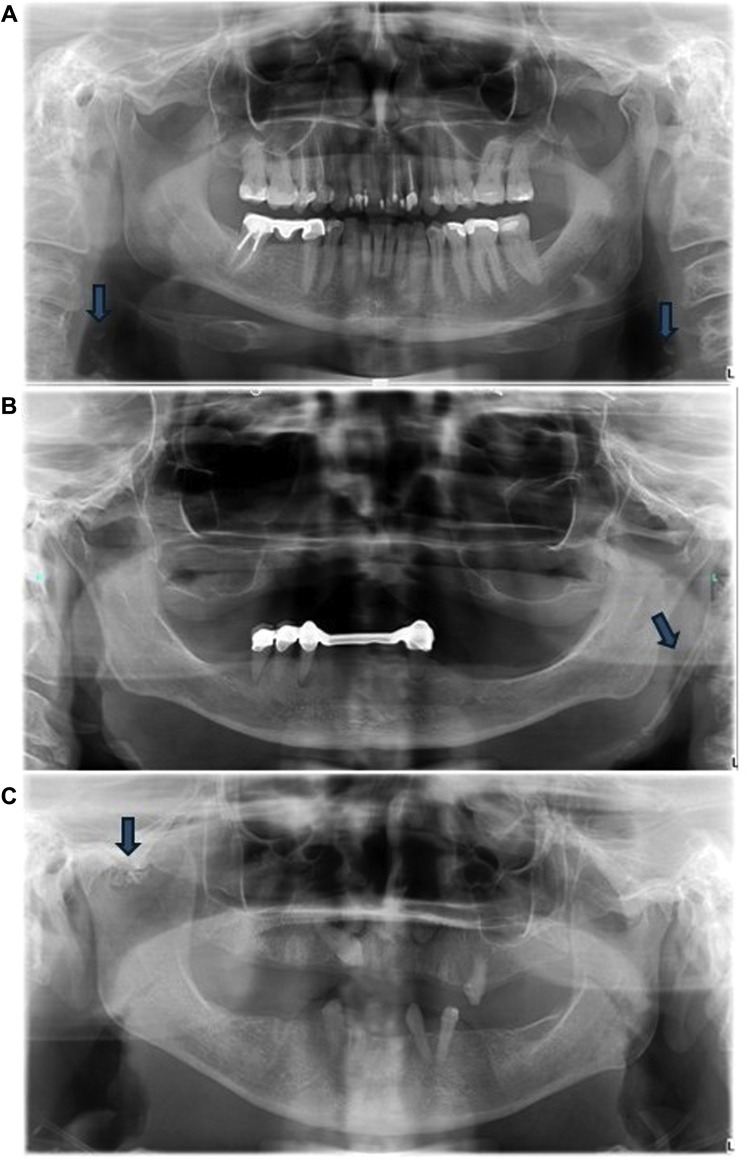
A, Representative panoramic radiographs showing soft tissue calcifications: suspected bilateral carotid-region calcifications, B, calcification/ossification of the stylohyoid ligament complex consistent with Eagle syndrome, and C, a phlebolith anterior to the right temporomandibular joint condyle.

For carotid-region calcification (potential carotid plaque), increasing age remained an independent predictor (per 10 years: aOR 1.52, 95% CI 1.08-2.14; *P* = .016). Diabetes status again showed a positive but nonsignificant association (aOR 1.62, 95% CI 0.66-3.94; *P* = .290), while sex, smoking, alcohol use, renal disease, and rheumatic disease were not significant contributors in the adjusted model.

For stylohyoid ligament complex calcification/ossification, no independent association was identified for diabetes (aOR 1.87, 95% CI 0.83-4.21; *P* = .134) or age (per 10 years: aOR 1.02, 95% CI 0.78-1.33; *P* = 0.901). The remaining covariates also did not reach statistical significance (Table 3).

**Table 3:** Multivariable logistic regression of panoramic radiographic calcification outcomes in the total study population.

Predictor	Any STC aOR (95% CI), *P*	Carotid-region aOR (95% CI), *P*	Stylohyoid Lg aOR (95% CI), *P*
Diabetes	1.55 (0.67-3.55); .303	1.62 (0.66-3.94); .290	1.87 (0.83-4.21); .134
Age (per 10 years)	**1.68 (1.23-2.29); .001**	**1.52 (1.08-2.14); .016**	1.02 (0.78-1.33); .901
Male sex	**0.40 (0.16-0.99); .047**	0.61 (0.23-1.63); .327	1.15 (0.48-2.74); .751
Smoking	1.74 (0.37-8.12); .481	1.12 (0.18-6.85); .902	0.45 (0.08-2.46); .359
Regular alcohol use	2.19 (0.34-13.98); .409	0.75 (0.07-7.51); .803	1.18 (0.18-7.74); .864
Renal disease	**0.17 (0.03-0.94); .042**	0.12 (0.01-1.15); .066	2.16 (0.48-9.66); .314
Rheumatic disease	0.68 (0.21-2.24); .526	1.39 (0.41-4.72); .597	2.39 (0.75-7.60); .139

In exploratory site-specific analyses, the prevalence of individual soft tissue calcification subtypes (including carotid-region calcifications, stylohyoid ligament complex calcification/ossification and tonsilloliths) did not differ significantly between the diabetes and control groups (all *P* > .05).

## Discussion

In this retrospective case–control study, participants were separated according to diabetes status and compared with an age- and sex-matched nondiabetic dental control group with highly similar baseline cardiometabolic profiles. The near-identical distribution of major comorbidities and lifestyle factors suggests that the control group constitutes a useful reference for the diabetes cohort and reduces the likelihood that measured baseline differences alone explain the radiographic findings.

Our findings should be interpreted in the context that PR provides opportunistic information on soft tissue calcifications; definitive evaluation of carotid atherosclerosis requires dedicated vascular imaging such as carotid ultrasound.

When the entire dataset was evaluated cross-sectionally, any soft tissue calcification, carotid-region calcification (potential carotid plaque), and stylohyoid ligament complex calcification/ossification were frequent incidental findings on PRs. In the fully adjusted models across the combined sample, age emerged as the most consistent independent correlate of both any soft tissue calcification and carotid-region calcification, while diabetes status did not retain an independent association after accounting for age, sex, smoking, alcohol use, renal disease, and rheumatic disease. This pattern indicates that, within the present dental cohort, the burden of radiographically visible calcification may be driven predominantly by age-related vascular and soft tissue changes, rather than by diabetes per se. The latter, is also supported by Griniatsos et al. who claimed that their study found a statistically significant lower incidence of diabetes mellitus, but a higher incidence of symptomatic plaques was observed in the group of patients with detectable calcifications on PR.^27^

A clinically important observation is that carotid-region calcification was common in both groups. Even though a statistically significant diabetes-related difference was not detected in the adjusted analyses, the overall frequency of suspected carotid-region calcifications warrants attention. Calcified atheromas in the carotid bifurcation region on PRs have been linked to ultrasonographically confirmed carotid plaque and stenosis and may reflect generalised atherosclerotic burden.^18^, ^19^, ^20^, ^21^, ^22^ Carotid plaque is a well-established predictor of future cerebrovascular events, and individuals with diabetes experience a substantially higher risk of ischaemic stroke and worse poststroke outcomes than those without diabetes.^1^ Therefore, our findings support the view that opportunistic recognition of cervical vascular calcifications on routine dental PRs may have value not only in patients with diabetes but also in nondiabetic dental patients with comparable cardiovascular risk profiles.

Our findings are in line with a broader body of evidence suggesting that oral and maxillofacial findings may serve as opportunistic indicators of systemic vascular and general health risk. Recent studies have reported associations between sublingual varices and heart- and cerebrovascular disease, and between occlusal support and long-term mortality in older adults.^28^^,^^29^ Together with these observations, the frequent detection of soft tissue calcifications on PRs supports the concept that oral examinations and dental radiographs can provide clinically useful information beyond the dentoalveolar region.

Within the combined sample, age emerged as the most consistent independent predictor of both any soft tissue calcification and carotid-region calcification, whereas male sex was associated with a lower likelihood of any soft tissue calcification. Diabetes status showed positive but statistically nonsignificant associations with all 3 outcomes, with aOR > 1 and wide confidence intervals that remain compatible with modest diabetes-related effects.

The absence of a clear diabetes signal in our adjusted models may have several explanations. First, the control group was intentionally selected to resemble the diabetes cohort in major cardiometabolic characteristics, which narrows between-group contrasts and can attenuate the independent contribution of diabetes. Second, the sample size, while sufficient for the primary design, may still limit power for detecting modest independent effects after multivariable adjustment. Third, PR is a 2-dimensional modality, and distinguishing among overlapping cervical soft tissue calcifications remains challenging; thus, some degree of nondifferential misclassification may have biased effect estimates toward the null. Against this background, the high carotid-region calcification prevalence in both cohorts should be interpreted as a signal for clinical vigilance, rather than as evidence against the relevance of diabetes in vascular risk.

The high prevalence of stylohyoid ligament complex calcification/ossification and its similar distribution between groups further suggests that this finding may represent a common incidental radiographic feature in middle-aged and older adults in a university dental setting. While such calcifications may be clinically silent, they can be associated with cervicofacial symptoms in a subset of patients, and their systematic reporting may support better differential diagnosis in routine care.

From a practical standpoint, our results reinforce the importance of structured reporting of soft tissue calcifications on PRs and of clear referral pathways. When calcifications in the carotid region are suspected, referral for medical evaluation and, where appropriate, carotid ultrasonography should be considered, especially in patients with multiple vascular risk factors. This is particularly relevant for individuals with diabetes, in whom stroke prevention remains a major clinical priority.^1^

### Limitations

This study has several limitations. Its retrospective, single-centre design may limit generalisability. We did not have systematic access to confirmatory ultrasound findings for all participants, which precluded direct validation of panoramic calcifications against a vascular imaging gold standard. Biochemical and anthropometric risk factors, such as lipid profiles or body mass index, were not uniformly available in the dental records. Finally, despite the use of multiple blinded examiners and consensus adjudication, some misclassification of calcification type or location is unavoidable with panoramic imaging.

The study was powered to detect a large between-group difference in soft tissue calcification prevalence (33% vs 11%); however, the observed difference was smaller (8.9%). Although aOR for diabetes were consistently > 1, wide confidence intervals indicate limited power, and the nonsignificant results do not exclude small-to-moderate diabetes-related associations.

This intentional selection of controls with similar comorbidity profiles, while likely attenuating between-group differences in calcification prevalence, was chosen to improve internal comparability in our case–control design and to isolate the potential added effect of diabetes.

### Strengths

Key strengths include the well-defined diabetes cohort derived from a specialised diabetes–dental collaboration, the carefully selected baseline-comparable control group, and the structured, multi-examiner radiographic evaluation. These elements strengthen the internal validity of the observed patterns and support the clinical relevance of the high overall burden of panoramic soft tissue calcifications.

## Conclusion

In summary, although diabetes status was not an independent predictor of the 3 calcification outcomes in the fully adjusted analyses, carotid-region calcifications were frequent in both groups. Given the established association between carotid atherosclerosis and stroke and the elevated cerebrovascular risk in diabetes, these findings highlight the potential role of routine dental PR as an opportunistic screening window for vascular risk markers, supporting closer interdisciplinary collaboration and timely medical referral when suspicious carotid-region calcifications are identified.^1^^,^^18^, ^19^, ^20^, ^21^, ^22^

## Ethics approval and consent to participate

The study was conducted in accordance with the Declaration of Helsinki and received approval from the Ethical Board of Semmelweis University under ethical clearance number RKEB: 204/2018.

## Data availability

The datasets used and/or analysed during the current study are available from the corresponding author on reasonable request.

## Author contributions

*Concept and design:* Végh, Bányai, Végh, Mühl. *Acquisition, analysis, or interpretation of data:* Lipták, Lipták, Mühl. *Drafting of the manuscript:* Lipták, Lipták, Mühl. *Critical revision of the manuscript for important intellectual content:* Hermann, Rózsa, Végh, Bányai, Kolarovszki, Marada, Végh. *Statistical analysis:* Lipták, Lipták, Mühl. *Supervision:* Hermann, Rózsa, Bányai, Kolarovszki, Marada, Végh.

## Funding

This research did not receive any specific grant from funding agencies in the public, commercial, or not-for-profit sectors.

## Conflict of interest

None disclosed.
